# 886 Incidence of Atrial Fibrillation in Burn Patients: A 6-Month Post-Burn Evaluation

**DOI:** 10.1093/jbcr/iraf019.417

**Published:** 2025-04-01

**Authors:** Zain Akbar, Rashid Syed, Abbas Karim, Suhaib Shah, Farhad Marzook, Juquan Song, Steven Wolf, Amina El Ayadi

**Affiliations:** University of Texas Medical Branch; University of Texas Medical Branch; University of Texas Medical Branch, John Sealy School of Medicine; University of Texas Medical Branch; University of Texas Medical Branch; University of Texas Medical Branch; University of Texas Medical Branch; University of Texas Medical Branch

## Abstract

**Introduction:**

Burns trigger complex physiological responses including inflammation and sympathetic activation, elevating the risk of cardiovascular complications. Atrial fibrillation is a common event in burn centers which can have ramifications on cardiac complications including death. We wondered whether new-onset atrial fibrillation has a significant relationship with diagnosis of heart failure in burned patients.

**Methods:**

De-identified electronic patient data were obtained from a large research network. The study population consisted of 13,187adult (>18 years) burned patients in the United States presenting with atrial fibrillation 6 months after injury; controls included 520,898 burned patients without atrial fibrillation.

**Results:**

About 3% of burn patients were diagnosed with new-onset atrial fibrillation. This population was 58% male and 70% Caucasian, with a mean age of 72±13 years.The mean heart rate post onset of atrial fibrillation was 79.4±19.4 bpm, while in those without atrial fibrillation it was 80.6±16 bpm. TBSA burned was similar in both groups. After propensity-matched with age, gender, race, ethnicity and burn severity of TBSA, we found that the atrial fibrillation group had a 9.247% in mortality and a 40.197% in heart failure (p< 0.05). The risk ratio for mortality for new-onset atrial fibrillation was 1.811, and for heart failure, 3.712 after propensity matching for age, gender, ethnicity, and %TBSA burned.

**Conclusions:**

Atrial fibrillation in burn patients is associated with significantly worse outcomes, including higher mortality and a dramatic increase in heart failure risk.

**Applicability of Research to Practice:**

Prevention and management of atrial fibrillation in burn patients is highlighted. Routine cardiovascular monitoring and targeted interventions are indicated.

**Funding for the Study:**

Database funding by the National Center for Advancing Translational Sciences

